# Definitive Obturator Fabrication for Partial Maxillectomy Patient

**DOI:** 10.1155/2020/6513210

**Published:** 2020-03-21

**Authors:** M. Singh, I. K. Limbu, P. K. Parajuli, R. K. Singh

**Affiliations:** Department of Prosthodontics and Crown-Bridge, B P Koirala Institute of Health Sciences, Dharan, Nepal

## Abstract

Maxillectomy defects can result in oroantral communication that causes difficulty in mastication and deglutition, impaired speech, and facial disfigurement. The prosthodontist plays an important role in the rehabilitation of such defects with obturators. This paper describes a clinical report of fabricating a definitive obturator with a cast metal framework using a single flask and one-time processing method for an acquired maxillary defect. A tripodal design was selected for this case. Rest was placed on the premolars and molars of both quadrants of the maxilla. Complete palate as the major connector was designed to ensure maximum distribution of the functional load to the tissue. Indirect retainer was planned on the right first premolar. Direct retention was provided by the I-bar clasp placed on the left first premolar, circumferential clasp on the right first premolar, and embrasure clasp between the right first and second molars. Thus, this definitive prosthesis rehabilitated the patient by providing better masticatory efficiency, improving the clarity of speech and quality of life of the patient.

## 1. Introduction

Palatal defects may result from congenital malformations, trauma, disease, pathologic changes, radiation burns, or surgical intervention [1]. These defects predispose the patient to hypernasal speech, leakage of fluid into a nasal cavity, and impaired masticatory function. Such defects need special prosthesis to establish oronasal seal which can be provided by obturator prosthesis [2]. The Glossary of Prosthodontic Terms defines an obturator as “a maxillofacial prosthesis used to close a congenital or acquired tissue opening, primarily of the hard palate and/or contiguous alveolar or soft tissue structures” [3]. The degree of obturator extension into the defect varies according to the configuration of the defect, character of its lining tissue, and functional requirements for stabilization, support, and retention of the prosthesis [4]. The open hollow obturator has disadvantages such as accumulation of food, debris, and mucus inside the hollow part leading to malodor and an increase in weight. The closed hollow obturator prevents water and food retention, enables cleaning, and has a reduced weight and maximum extension [5]. The size of the defect, number of remaining teeth, amount of remaining bony structures, and ability of the patient to adapt to the prosthesis are few factors that affect the prognosis of the treatment.

## 2. Case Report

**Figure 1:**
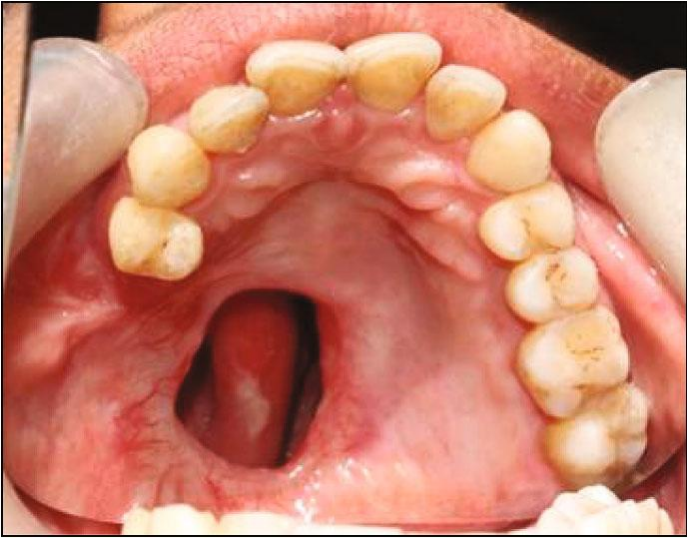
Intraoral defect of the palate.

A 38-year-old female presented to the Department of Prosthodontics and Crown-Bridge for the prosthetic rehabilitation of postmaxillectomy defect resulting from squamous cell carcinoma of the left maxilla 12 months back. The patient complained of difficulty in mastication, nasal regurgitation of fluids, and nasal tone in her voice. She had worn surgical and interim obturator. Intraoral examination revealed well-healed surgical defect in the maxilla involving part of the hard palate, alveolar ridge, and maxillary tuberosity creating an oroantral communication. All teeth posterior to the first premolar were missing on the left quadrant of the maxilla (Figure 1). Masticatory and phonetic functions of the patient were affected. After a thorough examination, the defect was classified as Aramany's Class II maxillary defect. The treatment plan was made to rehabilitate this patient with a definitive obturator with a cast metal framework.

## 3. Procedure

**Figure 2:**
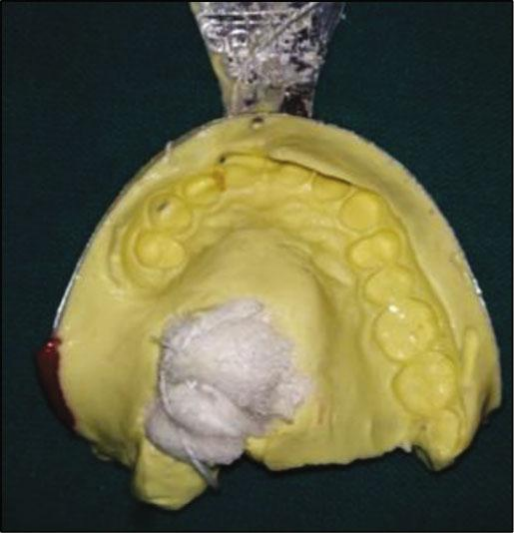
Primary impression.

**Figure 3:**
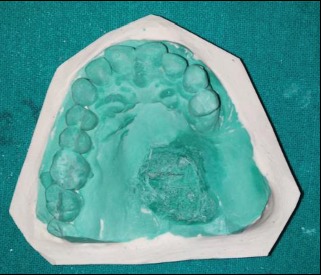
Primary cast.

The primary impression was made using irreversible hydrocolloid (Zelgan 2002, DENTSPLY) (Figure 2) and was poured with dental stone (Kalstone, Kalabhai) to obtain a primary cast (Figure 3). The defect was blocked with a gauze piece lubricated with petroleum jelly prior to impression making. The primary cast was then surveyed on a surveyor (MARATHON-Surveyor 103 Complete Milling Units # 100769), and the framework was designed. The design included a tripodal obturator design with complete palate as the major connector. Indirect retainer was planned on the right first premolar, and direct retention was provided by the I-bar clasp placed on the left first premolar, circumferential clasp on the right first premolar, and embrasure circumferential clasp on the right first and second molars. Rest seat preparations on 14, 16, 17, and 24 were carried out to receive rest of the cast metal framework following the principles of Aramany's Class II obturator design. Impression of preprosthetic mouth preparation was made with medium body elastomer (Reprosil, DENTSPLY), and the cast was poured with type IV dental stone (Kalstone, Kalabhai Karson).

**Figure 4:**
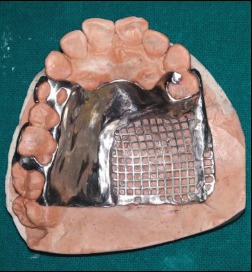
Metal framework of the obturator.

**Figure 5:**
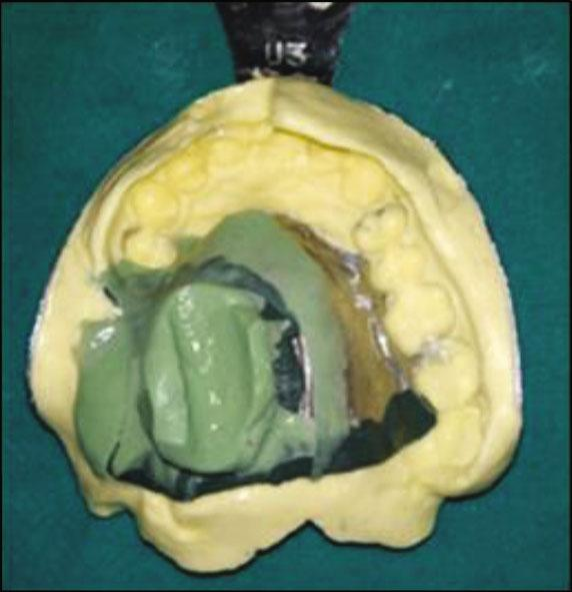
Pick-up impression.

**Figure 6:**
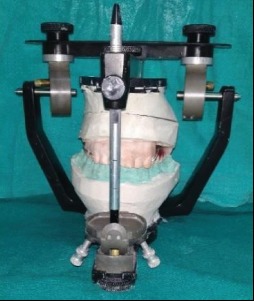
Teeth arrangement.

A tripodal configuration for the cast metal framework was designed. Designs of the cast metal framework were transferred on the cast, and a cast metal framework was fabricated and checked intraorally for fit and retention (Figure 4). Border molding was done using green stick impression compound (DPI pinnacle tracing sticks), and final impression of defect was made with light viscosity addition silicone impression material (Reprosil, DENTSPLY Caulk, USA). Pick-up impression was made over it with irreversible hydrocolloid (Zelgan 2002, DENTSPLY) and perforated stock tray (Figure 5). Master cast was poured and jaw relation was recorded and transferred to a semiadjustable articulator (Hanau Wide Vue Articulator) (Figure 6). Teeth were arranged on the metal framework, and wax try-in was carried out.

**Figure 7:**
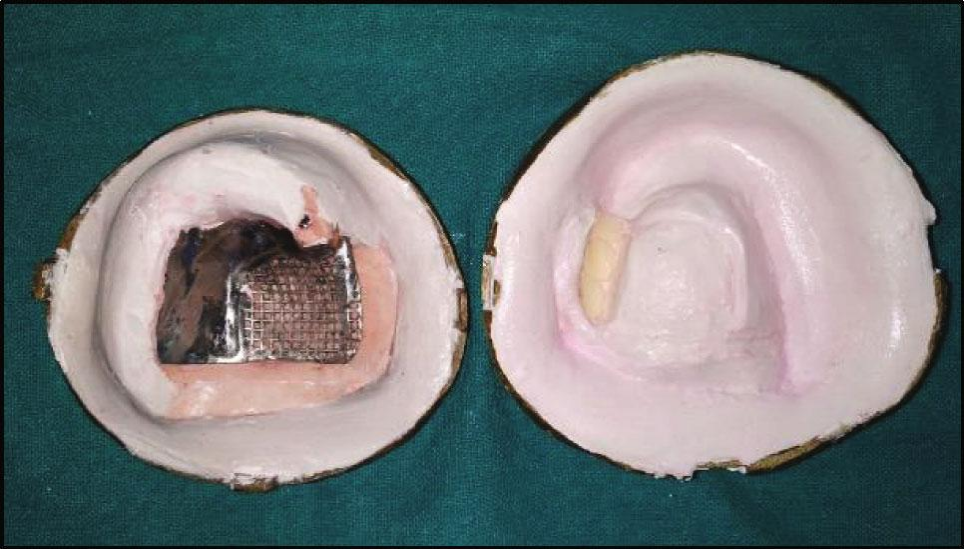
After dewaxing.

**Figure 8:**
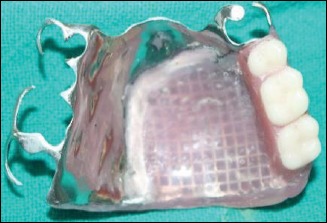
Occlusal view of prosthesis.

**Figure 9:**
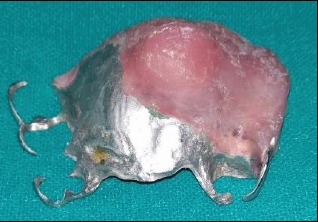
Impression view of prosthesis.

**Figure 10:**
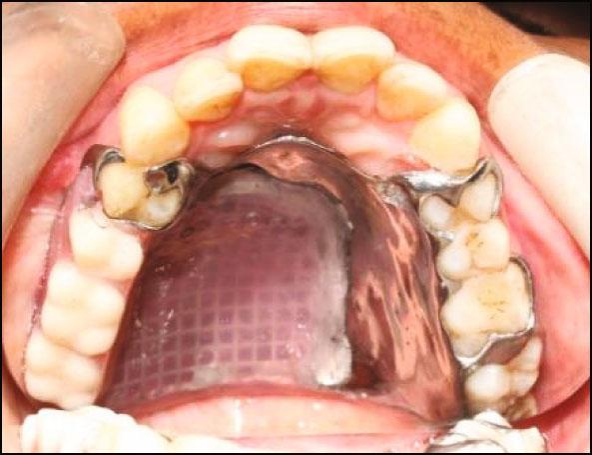
Prosthesis in situ.

**Figure 11:**
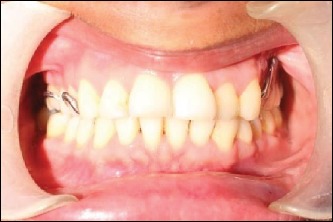
After prosthesis insertion.

After try-in, waxed up obturator was processed conventionally with flasking, dewaxing, and packing using heat-polymerizing acrylic resin (Trevalon Denture Material, DENTSPLY India Pvt. Ltd., India) (Figure 7). Finishing and polishing of the obturator prosthesis were done (Figures 8 and 9). It was then inserted into the patient's mouth after intraoral adjustments (Figures 10 and 11). The patient was happy and satisfied with her improved function, speech, and esthetics. The patient was instructed about the maintenance of the prosthesis and periodic recall check-up.

## 4. Discussion

Obturator prosthesis plays a crucial role in the recovery of oral function in postsurgical maxillectomy patients [1]. Framework designs for obturators may vary based on the classification system of the defect. All removable obturator prosthesis should be dictated by basic prosthodontic principles which include broad stress distribution, cross arch stabilization with the use of a rigid major connector, and stabilizing and retaining components at locations within the arch to best minimize dislodging functional forces [4].

A tripodal design was selected for this case. Support of the prosthesis was provided by the remaining teeth, palate, and rest. Rest was prepared on the right and left first premolars and first and second molars of the right quadrant of the maxilla. Complete palate was designed to ensure maximum distribution of the functional load to the tissue. Indirect retainer was planned on the right first premolar. Direct retention was provided by the I-bar clasp placed on the left first premolar, circumferential clasp on the right first premolar, and embrasure circumferential clasp between the right first and second molars [6–8].

In dentate patients, the remaining teeth play an important role in providing retention, support, and stability to the obturator. Retention can be achieved from the remaining teeth or ridge, lateral part of the defect, soft tissue undercut, and scar band. Stabilization and indirect retention components must be positioned effectively to retard the movement of the defect extension portion away from its terminal position [9].

Different types of retentive aids such as magnets, snap-on (friction-type) attachments, acrylic buttons, retentive clips, and implants are used for the conventional obturator prosthesis. The use of implant is a new advancement in maxillofacial prosthodontics. They effectively improve the retention of prosthesis without the help of other appliance. However, cost, health of the patient, and bone qualities are some of the factors which limit the use of implants [10].

The advantages of metal framework obturator prosthesis are the longevity of the prosthesis and thermal conductivity of metal which made it sensitive to temperature change [4, 5].

## 5. Conclusion

The great challenge in rehabilitating a hemimaxillectomy patient is to obtain adequate retention, stability, and support. Thorough knowledge and skills coupled with a better understanding of the needs of the patients enable the successful rehabilitation of such patients. Definitive obturator prosthesis fabricated with maximum extension and proper design rehabilitates the patient by improving masticatory efficiency, increasing the clarity of speech and quality of life.
